# Epitranscriptomic Signatures in lncRNAs and Their Possible Roles in Cancer

**DOI:** 10.3390/genes10010052

**Published:** 2019-01-16

**Authors:** Sorina Dinescu, Simona Ignat, Andreea Daniela Lazar, Carolina Constantin, Monica Neagu, Marieta Costache

**Affiliations:** 1 Department of Biochemistry and Molecular Biology, University of Bucharest, 050095 Bucharest, Romania; simona.rebeca.ignat@drd.unibuc.ro (S.I.); andreealazar12345@gmail.com (A.D.L.); marieta.costache@bio.unibuc.ro (M.C.); 2 Immunology Department, “Victor Babes” National Institute of Pathology, 050096 Bucharest, Romania; caroconstantin@gmail.com (C.C.); neagu.monica@gmail.com (M.N.)

**Keywords:** lncRNAs, chemical modifications, MALAT1, HOTAIR, XIST, epitranscriptomics, cancer

## Abstract

In contrast to the amazing exponential growth in knowledge related to long non-coding RNAs (lncRNAs) involved in cell homeostasis or dysregulated pathological states, little is known so far about the links between the chemical modifications occurring in lncRNAs and their function. Generally, ncRNAs are post-transcriptional regulators of gene expression, but RNA modifications occurring in lncRNAs generate an additional layer of gene expression control. Chemical modifications that have been reported in correlation with lncRNAs include m^6^A, m^5^C and pseudouridylation. Up to date, several chemically modified long non-coding transcripts have been identified and associated with different pathologies, including cancers. This review presents the current level of knowledge on the most studied cancer-related lncRNAs, such as the metastasis associated lung adenocarcinoma transcript 1 (MALAT1), the Hox transcript antisense intergenic RNA (HOTAIR), or the X-inactive specific transcript (XIST), as well as more recently discovered forms, and their potential roles in different types of cancer. Understanding how these RNA modifications occur, and the correlation between lncRNA changes in structure and function, may open up new therapeutic possibilities in cancer.

## 1. Introduction

Long non-coding RNAs (lncRNAs) have recently been recategorized from ‘junk’ non-coding material to master regulators of transcriptional, post-transcriptional, and translational levels of expression. LncRNAs are a category of non-coding RNAs with distinctive features and exhibit tissue specificity. These molecules display lengths between 200 nt and 100 kilobases (kb), and originate from the non-protein coding regions of the genome. LncRNAs resemble mRNA-like transcripts, since they possess a CAP structure, polyadenylated tail, and are transcribed by RNA polymerase II (RNA Pol II). However, these transcripts lack conserved open reading frames [1].

To date, studies have shown that lncRNAs are involved in up- and down-regulation of gene expression at both transcriptional and post-transcriptional levels in all fundamental cellular processes—proliferation, differentiation, development, immunity, altered metabolism, and signaling, including in cancer states [2,3]. Although they have started to be intensively studied in the last years, insufficient information is known so far about lncRNA mechanisms of action. LncRNAs can act as guides and chromatin regulators [4], scaffolds for ribonucleoprotein complexes [5], decoys or enhancers for transcription factors [1], and sponges for other post-transcriptional regulators such as microRNAs (miRNAs) [6].

Cancer is a dysregulated state, characterized by chaotic signaling and abnormal patterns of expression resulting from multiple genetic and epigenetic alterations, which have been frequently associated with ultra-conserved, non-coding sequences of the genome [1]. These abnormalities also affect lncRNAs, disrupting their functions and consequently leading to deregulation of their targets [7]. As ’fine tuners’ of either tumor suppression or tumorigenesis [7], lncRNAs are reported to be involved in different phases of cancer development and progression. Calle et al. recently discussed a lncRNA code in cancer, and managed to classify these lncRNAs into two categories: tumor-suppression-associated lncRNAs (e.g., LED, Linc-p21, GUARDIN, PTENP1) and tumor-promotion-associated lncRNAs (e.g., MALAT1, HOTAIR, NORAD, PVT1). Furthermore, due to their tissue specificity, these cancer-related lncRNAs have the potential to become diagnostic and prognostic markers, as well as potential targets for innovative therapeutic strategies.

Recent research and evolution of detection methods has led to the identification of several dynamic chemical modifications in RNAs that have been shown to modulate their activity, and therefore can be considered ‘switches’ [8,9]. Nevertheless, these switches are controlled by enzymes that produce the modifications (‘writers’), complexes that recognize the change and bind to the new modified motif (‘readers’), and enzymes that are able to remove the modified base and regress RNA to the original structure (‘erasers’). The existence of erasers (e.g., demethylases) and the interplay between writers and erasers (e.g., methylation–demethylation) means that the chemical modifications are dynamic and reversible, and the enzymes that govern these switches are highly evolutionarily conserved [9]. To date, more than 160 chemical modifications in RNA have been documented [9], out of which more than half were found to be involved in human diseases, including cancer [10].

The aim of this review is to explore the cancer-related lncRNA landscape, and to highlight to readers the interplay between the dynamic chemical modifications that may occur within lncRNAs’ structure, and the effect they may have on their function. Overall, these epitranscriptomic changes lead to modified lncRNAs that generate an additional layer of post-transcriptional control, having a great impact on human health and disease.

## 2. Chemical Modifications Occurring in Long Non-Coding RNAs

Post-transcriptional modifications that occur in RNA molecules started being explored recently, giving rise to a new field of research called epitranscriptomics. Equivalent to epigenetics, which analyzes post-transcriptional molecular events occurring in DNA, epitranscriptomics investigates modifications resulting from all RNA processing events, such as RNA splicing, RNA editing, or methylation [11]. Among these modifications, the most frequent and well described are N^6^-methyladenosine (m^6^A), N1-methyladenosine (m^1^A), 5-methylcytosine (m^5^C), pseudouridine (Ψ), and adenosine to inosine transition (A-to-I) [12,13]. According to Esteller et al. [14], described chemical modifications in lncRNAs include m^5^C, m^6^A, Ψ, CAP, splicing, editing, and U-tail. Next, the most common modifications will be further described in detail, showing their specific writers, erasers, and readers.

### 2.1. N^6^-Methyladenosine (m^6^A)

N^6^-methyladenosine (m^6^A) is the most abundant and the first internal modification of messenger RNA [15]. This RNA modification can also be found in non-coding RNAs (ncRNAs) such as small nucleolar RNAs (snoRNAs), transfer RNAs (tRNAs), ribosomal RNAs (rRNAs), and lncRNAs, and it affects several RNA related processes: RNA splicing, translation, and stability [16,17]. One messenger RNA (mRNA) can contain up to 5 m^6^A sites, adding up to between 0.1–0.4% modified sites in mRNA [18,19,20]. The m^6^A sites are frequent around stop codons, in 3’-untranslated region (UTR), and in long exons [21,22]), and there is one consensus motif in which m^6^A is mostly found: DRACH sequence (D = A/G/U, R = A/G, H = A/C/U) [19,23]. The reversibility of the m^6^A modification was discovered in 2011, when the first RNA demethylase for m^6^A was identified [24]. The m^6^A dynamics are modulated by the action of a few regulators organized in ‘writers’ (methyltransferases), ‘readers’ (binding proteins), and ‘erasers’ (demethylases) [25].

The m^6^A modification is catalyzed by a complex of RNA methyltransferases (‘writers’) which includes one heterodimer, the methyltransferase-like 3 (METTL3)/METTL14, and other cofactors [26,27]. The methyltransferase activity of the complex is improved by the METTL14 association to METTL3, due to the role of METTL14 in substrate binding [25,28,29].

Another component of the m^6^A writer complex is Wilms’ tumor 1-associating protein (WTAP), which binds both METTL3 and METTL14 and ensures their recruitment to nuclear speckles [30]. WTAP also shows potential to bind other proteins and lncRNAs as well, increasing the number of potential other factors recruited to the complex [25,31]. KIAA1429 or vir like m^6^A methyltransferase associated (VIRMA) is also part of the complex and influences methylation around stop codons and in UTR. Depletion of KIAA1429 leads to a lower number of m^6^A sites in RNA [32,33].

Among other adaptor proteins that interact with WTAP are RNA-binding motif protein 15 (RBM15) and its paralogue RBM15B [31,32,34]. They mediate the binding of the writer complex to specific sites. One lncRNA that represents a target for RBM15 is the X-inactive specific transcript (XIST). Loss of RBM15/RBM15B expression leads to fewer m^6^A sites [34].

METTL16 is a U6 small nuclear RNA (snRNA) methyltransferase with independent action from the METTL3 complex. METTL16 is responsible for methylation within the UACAGAGAA motif in U6 snRNA. METTL16 is also involved in S-adenosyl methionine (SAM) homeostasis control, by methylating inside an intron of methionine adenosyltransferase 2A (MATA2A) [35]. Another role of METTL16 is to bind the 3’ terminal triple helix of lncRNA metastasis associated lung adenocarcinoma transcript 1 (MALAT1) [36].

The m^6^A binding proteins, also known as ‘readers’, are either direct readers or indirect readers. This distribution indicates the RNA-binding method [26,37]. The direct readers associate to specific RNA m^6^A sites. The indirect readers are able to bind to RNA as a result of an RNA secondary structure relaxation induced by m^6^A modification [38,39].

The proteins that belong to the YT521-B homology domain family (YTHDF) are direct readers with cytoplasmic (YTHDF1-3 and YTHDC2) or nuclear (YTHDC1, YTHDC2) distribution. While YTHDF1 regulates RNA translation [40], YTHDF2 binds mRNA and ncRNA that contains m^6^A, and affects their stability by inducing their degradation [41]. YTHDF3 is capable of regulating the RNA-binding of either YTHDF1 or YTHDF2, these three proteins composing a regulatory mechanism for the metabolism of m^6^A-containing RNA [25]. YTHDFC1 controls pre-mRNA splicing [42] and also the processing of certain ncRNAs, such as XIST. YTHDFC2 localizes to the cytoplasm and the nucleus. Along with YTHDFC2’s role in mRNA translation, this protein binds to m^6^A-containing lncRNAs [25,34].

Heterogeneous nuclear ribonucleoprotein A2/B1 (HRPNPA2B1) is another direct reader involved in miRNA processing [43]. Heterogeneous nuclear ribonucleoprotein C (HNRNPC) is an indirect reader of m^6^A, which opposes poly-U hairpin RNA [39].

The m^6^A modification can be reversed through the action of two m^6^A demethylases (‘erasers’) that belong to the Fe(II)/α-KG-dependent dioxygenase AlkB family: the mass- and obesity-associated protein (FTO) and α-ketoglutarate-dependent dioxygenase alkB homolog 5 (ALKBH5) [25]. Although FTO was the first m^6^A eraser discovered [24], following investigations suggest its action may target another very similar adenosine modification, N^6^ 2’-O-dimethyladenosine (m^6^A m) [44]. However, the proposed course of action for FTO was that it induces the oxidation of m^6^A to N^6^-hydroxymethyladenosine (hm^6^A), and further to N^6^-formyladenosine (f^6^A) [45]. Demethylation induced by FTO was shown to promote adipogenesis [46] and cap-independent translation [47]. Overexpression of FTO in some acute myeloid leukemia (AML) also affects the m^6^A levels in certain mRNA transcripts that further promote leukemogenesis [48].

Knockdown of *alkbh5* in mice affects mouse fertility and spermatogenesis, and in humans affects nascent mRNA synthesis and the rate of splicing [49]. Hypoxia-induced ALKBH5 expression in breast cancer cells enhances mRNA stability of homeobox transcription factor NANOG and induces its overexpression, leading to a phenotype specific to breast cancer stem cells [50]. Another role of ALKBH5 is in glioblastoma stem-like cells (GCSs). ALKBH5 demethylates nascent Forkhead box protein M1 (FOXM1) transcripts, leading to increased FOXM1 expression, a factor involved in GSC proliferation. It has been shown that by ALKBH5 inhibition, the tumorigenesis of GCSs is also repressed [51].

### 2.2. N^1^-Methyladenosine (m^1^A)

Although not as abundant as m^6^A, another important RNA modification is N^1^-methyladenosine (m^1^A) [52]. Initially, this type of modification was identified only in ncRNAs, typically found in the tRNA T-loop, but its presence in mRNA was recently confirmed as well [16,53,54]. It is usually located within the 5’-untranslated region (5’-UTR) and highly structured regions [53,54]. Under non-physiological conditions, m^1^A is charged positively, which can affect the structure of RNA and the protein–RNA interactions. The role of m^1^A modification has not been completely elucidated, but it is suggested that it promotes protein production and a more efficient translation process [54].

The writers for m^1^A are tRNA m^1^A methyltransferases (MTase), and they are different for each specific m^1^A location [55,56,57]. Human nucleolar protein nucleomethylin (NML) is responsible for the addition of two m^1^A in rRNA [58], and in mitochondrial transfer RNA (mt-tRNA) m^1^A is catalyzed by tRNA methyltransferase 10 C, mitochondrial RNase P subunit (TRMT10C), and tRNA methyltransferase 61B (TRMT61B) [59,60]. Usually, in mRNA, TRMT6/61A recognizes a consensus GUUCRA motif within the tRNA loop-like structure. As for lncRNAs, the specific writers have not been identified, but many sites with the same GUUCRA motif can undergo m^1^A modification. MALAT-1, a lncRNA which is highly expressed in many types of cancer, can also suffer m^1^A modification in A8398 position [56,61,62].

The readers for m^1^A sites are mostly the same as those for m^6^A modification: YTHDF1, YTHDF2, YTHDF3, and YTHDC1 [63].

An important aspect of m^1^A modification is its reversibility, and there are 2 erasers identified that ensure m^1^A demethylation, ALKBH3 and ALKBH1 [53,64]. High levels of ALKBH3 are linked with some human cancers, namely angiogenesis in prostate cancer and pancreatic cancer, and it has been shown to inhibit apoptosis [65,66].

### 2.3. 5-Methylcytosine (m^5^C)

Although early studies demonstrated the existence of 5-methylcytosine (m^5^C) in mRNA, tRNA, and viral RNA, it has only recently been thoroughly characterized in RNA, as it was considered to be primarily a modification of DNA [67,68,69]. When present, m^5^C is positioned about 100 nucleotides downstream of the translation initiation site in human and murine mRNAs, and it can also be found in the UTRs [70,71,72]. It also appears in rRNA and lncRNA [73].

There are two groups of m^5^C writers: the NOP2/SUN RNA methyltransferase (NSUN) family, with seven members, and DNA methyltransferase-2 (DNMT2), previously thought to methylate DNA. The substrate(s) for each member of NSUN family, as well as DNMT2, is mentioned in Table 1 [74,75,76,77,78,79].

Mutations in *Nsun* genes lead to different diseases, for example, alteration of *Nsun7* in mice causes sperm motility problems, with various grades of infertility, while mutations in *Nsun2* are associated with autosomal-recessive intellectual disability [80,81]. Overexpression of *Nsun2* by hypomethylation is present in human cancers, and is associated with metastatic progression in human breast cancer [82,83]. Altered expression levels of *DNMT2* have also been observed in human malignant cells [84]. More than 60 somatic mutations have been detected in hundreds of tumor samples, data collected by the Catalogue of Somatic Mutations in Cancer (COSMIC) database [85].

A recent study suggested the Aly/REF export factor (ALYREF) as a reader for m^5^C. This m^5^C binding protein promotes selective mRNA export from the nucleus, suggesting a potential role for m^5^C in RNA transport. The full functions of m^5^C remain to be discovered [86].

While there is no known protein that can fully determine the regression of m^5^C to cytosine, m^5^C can be ‘erased’ and turned into 5-hydroxymethylcytosine (hm^5^C) by the ten-eleven family demethylases (TET) that also direct DNA demethylation [87,88].

### 2.4. Pseudouridine (ψ)

Also called ‘the fifth nucleotide’ due to its high abundance, 5-ribosyluracil or pseudouridine (ψ) was discovered in 1951 and is the most predominant RNA modification, found in all types of RNA from mRNA to ncRNAs, such as rRNA, tRNA, snRNA, snoRNA, and lncRNAs [89,90]. It is formed through isomerization of uracil, with the C1’ of the ribose binding to uracil’s C5, which frees N^1^ and enables it to form additional hydrogen bonds, leading to a more rigid sugar–phosphate backbone and enhanced folding. Although ψ binds to adenosine in the same manner as uridine, its interaction with the other bases is stronger [91,92].

According to recent studies, pseudouridylation can also affect mRNA’s coding potential. In yeast, the presence of ψ in stop codons suppressed translation termination by guiding the incorporation of new amino acids [93,94]. The distribution pattern of ψ can also be altered by stress (heat, oxidative stress, nutrient deprivation), as observed in yeast and human cells [95,96].

The enzymes that catalyze the conversion of uridine to ψ, the so called ‘writers’, are pseudouridine synthases (PUSs). Their action can be conditioned or not by the presence of RNA, resulting into two categories: RNA-dependent and RNA-independent. The RNA-dependent PUSs associate with cofactors and the H/ACA box of snoRNAs, forming a complex that interacts with the RNA target in a site-specific manner based on sequence complementarity, e.g., H/ACA ribonucleoprotein complex subunit 4 (dyskerin) [97]. This means that the RNA-dependent PUSs need other small RNA molecules to guide them to their target, while independent PUSs have no need of them and can perform their catalytic function without these intermediary RNAs. Independent PUSs often bind to conserved structural or specific sequence motifs of the target. For example, pseudouridylation of ncRNAs primarily occurs within paired structures [98,99].

Unfortunately, specific ‘readers’ and ‘erasers’ remain unknown. The absence of an eraser protein could be explained by the fact that the C–C bond formed between the base and the ribose in ψ is much more inert than the C–N bond in uridine, possibly making pseudouridylation irreversible [100].

### 2.5. Strategies and Methods Currently Used to Detect Modifications

To date, the major technology used for the identification of dynamic RNA modifications relies on sequencing. However, there are some important downfalls of this technology. Next, we will present major sequencing variants developed for detection of each chemical modification, and their pro and cons. Last but not least, computational tools that identify modification sites from sequencing data are still under development.

A transcriptome-wide profile of m^6^A can be achieved with m^6^A-seq or methylated RNA immunoprecipitation sequencing (MeRIP-seq), two separate methods based on m^6^A-specific methylated immunoprecipitation, followed by NGS. After isolation, poly(A)-enriched RNA is fragmented to 100–150 nt and immunoprecipitated with m^6^A-specific antibodies. Libraries are constructed from the fragments that contain m^6^A, taking as controls non-immunoprecipitated samples, and subjected to high-throughput-sequencing. Although easily manageable, these methods have two significant disadvantages: slightly low resolution (100–200 nt) and antibody specificity (they also recognize m^6^A m), making identification of specific m^6^A sites at single nucleotide level difficult [101,102].

Photo-crosslinking-assisted m^6^A sequencing (PA-m^6^A-seq) was developed in order to overcome some of the difficulties presented by m^6^A-seq and MeRIP-seq [103]. This strategy facilitates detection of m^6^A at single-base resolution due to the incorporation of photoactivatable ribonucleoside 4-thiouridine (4-SU) in RNA. After m^6^A immunoprecipitation, the antibody is crosslinked to 4-SU under 365 nm UV light and the crosslinked RNA is fragmented by RNase T1 to about 30 nt, after which sequencing commences. During reverse transcription-polymerase chain reaction (RT-PCR), at the site of crosslinking, 4-SU determines a U/T to C transition, resulting in better signal-to-noise ratio of methylation detection. However, not all m^6^A modifications have a nearby site for 4-SU incorporation, and some m^6^A sites may be missed [103].

The methylation status of a single modified nucleotide can be quantified with site-specific cleavage and radioactive-labelling followed by ligation-assisted extraction and thin-layer chromatography (SCARLET) [104]. This method is not restricted to m^6^A detection, it can also be used for identification of m^5^C and ψ [96]. In SCARLET, the candidate site is flanked by sequence-specific probes that act as a guide for RNase H. After the site-specific cleavage, the resulting RNA fragments are radiolabeled and splint-ligated to a single-stranded DNA oligonucleotide. The samples go through RNA digestion, gel purification, and Nuclease P1 treatment before being separated by thin layer chromatography [104].

Two methods (m^1^A-seq and m^1^A-ID-seq) have been used to carefully map m^1^A modification in the eukaryotic transcriptome [53,54]. They resemble the m^6^A detection methods (m^1^A-seq is an adapted version of MeRIP-seq), as they also rely on the combination of immunoprecipitation and high-throughput-sequencing. Both of them have succeeded in generating high resolution maps, due to different strategies. While in m^1^A-seq an m^1^A-to-m^6^A rearrangement occurs under alkaline conditions, turning RT-interfering m^1^A to RT-silent m^6^A, in m^1^A-ID-seq an RNA/DNA demethylase reverts the modified base to regular A after immunoprecipitation. Peaks of m^1^A are detected by comparing the NGS reads of the demethylase treated and untreated fragments [53].

For the detection of widespread m^5^C modifications there are three major techniques: (a) detection at single nucleotide resolution through bisulfite conversion, also called bisulfite sequencing (bsRNA-seq), (b) 5-azacytidine-mediated RNA immunoprecipitation (Aza-IP), and c) methylation individual-nucleotide-resolution cross-linking and immunoprecipitation (miCLIP) [105].

Bisulfite treatment determines the conversion of unmodified cytidine to uridine (read as thymidine during sequencing), while m^5^C remains unchanged. This difference is detected by Sanger sequencing or by next-generation sequencing (NGS) after library preparation [73,106]. However, bsRNA-seq has some limitations. For instance, in double-stranded RNA regions cytosine might remain unaffected by the treatment with bisulfite, which will lead to a false positive result for m^5^C in those regions. The same goes for other modifications of cytosine other than m^5^C, which will most likely be resistant to the treatment as well and misidentified as m^5^C sites [105]. These drawbacks can be overcome by immunoprecipitation of fragmented RNA with m^5^C-specific antibody or a control antibody prior to sodium bisulfite treatment (the total RNA will firstly be depleted of rRNA and enriched with polyA), followed by library construction and NGS [107].

The second method, Aza-IP, relies on random incorporation of the anti-cancer drug 5-azacytosine into the nascent RNA of cells that overexpress affinity-tagged RNA methyltransferases (RMTs), followed by immunoprecipitation of those tagged enzymes and NGS. The incorporated 5-azacytidine acts as a suicide substrate because of the covalent bond that is formed between it and m^5^C-RMTs. The RNA targets are isolated and identified by affinity purification of the tagged RMTs, partial RNase digestion, and RNA sequencing, with the resulting fragments being used for the construction of a cDNA library and finally NGS. During sequencing, modified cytidine residues are read as guanidine instead of normal cytidine. This facilitates the clear identification of the modified nucleotides. Direct targets of DNMT2 and NSUN2 could be identified using this technique [71,108].

The third method, called miCLIP, is a derivative of individual-nucleotide-resolution cross-linking and immunoprecipitation (iCLIP) method, and it relies on the use of an overexpressed affinity-tagged mutant RMT. This approach has been used before to asses NSUN2’s specific sites of methylation [109]. In this case, C271A (a mutant of NSUN2) formed a stable link with its target cytosine residue and the complex was immunoprecipitated. Afterwards, m^5^C mapping with NGS commenced. During library preparation, reverse transcription terminated at the cross-link site of the cytosine with the modified protein, resulting in a high cytosine appearance in position +1 in cDNA libraries (the first nucleotide to be read), allowing for m^5^C detection at single nucleotide resolution [110,111]. Using this technique, new mRNA and ncRNA transcripts were identified as methylation targets, for example vault RNAs (found in cytoplasmic ribonucleoprotein complexes of unknown function termed vault particles) [112]. The miCLIP method also serves as a detection method for m^6^A residues [23].

As an isomer of uridine, mapping ψ in a site-specific quantitative manner can be rather challenging. Nevertheless, there are some methods we can employ in order to detect ψ, some more successful than others. One method consists of labelling ψ with N-cyclohexyl-N9-(2-morpholinoethyl)-carbodiimide metho-p-toluenesulphonate (CMCT), followed by alkaline hydrolysis which removes adducts formed with U or G, leaving behind only CMCT-ψ. This CMCT-ψ terminates reverse transcription one nucleotide downstream of the pseudouridylated positions, resulting in short cDNA fragments that are used for library preparation. NGS is performed for cDNA libraries constructed with or without CMCT treatment, and ψ is mapped by calculating the differences between the stop rates of the two samples [113,114,115]. This method was independently used by several groups, and each of them came up with different names for it: ψ-seq, Pseudo-seq, or PSI-seq for yeast [95,116,117].

Increased detection sensitivity was achieved with the adaptation of this approach developed by Li et al. [96]. They named it N3-CMCT-enriched pseudouridine sequencing (CeU-seq). A derivative of CMCT is used, which not only marks ψ, but it allows the formed CMCT-ψ to be labelled with DBCO-(PEG)4-biotin by click chemistry, followed by immunoprecipitation with streptavidin beads, reverse transcription, and sequencing. This leads to more modified RNA molecules that non-modified ones, which gives a better signal to noise ratio, resulting in the detection of small quantities of ψ-RNA modified transcripts [96].

There are some other strategies employed apart from these sequencing-based methods, for example SCARLET, a good validating method for base modifications and determination of the stoichiometry [96,104]. Another approach involves breaking down RNA into nucleosides by enzymatic hydrolysis, followed by liquid chromatography and mass spectrometry for identification. Unfortunately, there is no difference between uridine and ψ, and therefore ψ must be marked with chemical labels [118].

From the methodological point of view we are just starting to comprehend the need to increase the resolution and sensitivity of these methods. High-throughput sequencing techniques generate huge amounts of data that need further validation with new approaches. Combinations of third-generation sequencing, new chromatography methods, and new mass spectrometry approaches will improve studies in the field of epitranscriptomics.

## 3. Long Non-Coding RNAs with Activity in Cancer

**Figure 1:**
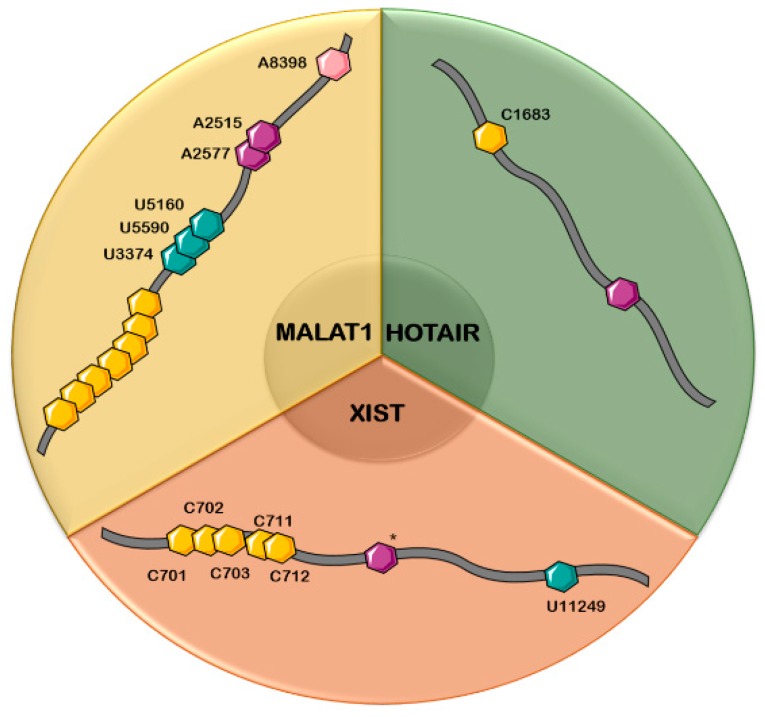
RNA modifications occurring in the main long non-conding RNAs (lncRNAs) and their positions: MALAT1, HOX antisense intergenic RNA (HOTAIR), and X-inactive specific transcript (XIST). *Different numbers of m^6^A sites are reported in XIST (presented in Table 2). Blue hexagon—ψ modification, purple hexagon—m^6^A modification, yellow hexagon—m^5^C modification, pink hexagon—m^1^A modification.

There are several lncRNAs involved in different types of cancers, which simultaneously acquire one or more dynamic modifications within their structures (Table 2). Among these lncRNAs, XIST, MALAT1, and HOTAIR will be presented in detail below, along with their respective RNA modifications known to date (Figure 1).

### 3.1. X-Inactive Specific Transcript

The X-inactive specific transcript (XIST) is a lncRNA (17–20 kb) involved in the permanent inactivation of one of the two X chromosomes, an early developmental process in mammalian females. XIST is expressed from a specific region termed the X inactivation center (XIC), and only from the future inactive X chromosome. Once transcribed, XIST is not translated, instead accumulating in the nucleus where it coats the inactive X chromosome in cis. This transcriptional silencing mechanism provides dosage equivalence between males and females [119,120,121,122].

#### 3.1.1. Chemical Modifications in X-Inactive Specific Transcript

In order for X inactivation to take place, a region composed of 8.5 repeats (R1–R8.5) with 26 nt per full repeat from the 5’-end of XIST, called the repeat A-region, associates with the polycomb-repressive complex 2 (PRC2), a chromatin-associated protein complex [119]. Amort et al. identified a cluster of m^5^C within repeat 8 of this region, using a protocol they developed based on bisulfite treatment and PCR amplification of poly(A)-enriched RNA. The analysis revealed five methylated cytosines in positions 701–703, 711, and 712 for 19–24% of the amplicons, while simultaneous methylation of all five residues was found in 19% of the sequences [123]. However, we cannot talk about a conserved mechanism, since no methylation was observed at the corresponding cytosines (668–670, 678) in murine sequences. Nonetheless, the methylated sites affected the binding properties of R8 to the PRC2 complex, indicating that posttranscriptional modifications of cytosine can modulate XIST–protein interactions [123].

Another chemical modification, specifically a ψ residue in U11249, was discovered by Li et al., but the impact of this structural change on the function of XIST is currently unknown [96].

It has been reported that, in human cells, XIST is methylated with no less than 78 m^6^A residues, and this methylation promotes XIST-mediated transcriptional repression [34]. The formation of m^6^A in adjacent consensus motifs relies on the interaction between m^6^A-methylation complex and two RNA-binding proteins, RBM15 and its paralogue RBM15B, that bind the complex to XIST. The methylated sites are recognized by YTH domain containing 1 (YTHDC1) protein and gene silencing occurs, although it is not clear how. Knockdown of RBM15, RBM15B, or methyltransferase METTL3 inhibits X-mediated gene silencing [34].

#### 3.1.2. X-Inactive Specific Transcript in Cancer

X-inactive specific transcript is one of the best studied lncRNAs, and as such it has been searched for and found in many different human neoplasias. Its expression can either be upregulated or downregulated, acting as an oncogene or as a suppressor in multiple types of cancer. Overexpression of XIST is associated with advanced tumor stage, lymph node or distant metastasis, and overall poor prognosis in human cancers [124].

In breast cancer, XIST acts as a tumor suppressor by positively regulating the expression of non-X-chromosome gene PH domain and leucine rich repeat protein phosphatase 1 (PHLPP1), which in turn catalyzes dephosphorylation of protein kinase B (AKT) [125]. In non-small-cell lung cancer (NSCLC), nasopharyngeal and hepatocellular carcinoma, gastric, colorectal, pancreatic, bladder cancer, and osteosarcoma, its expression is upregulated and this lncRNA plays the role of an oncogene, promoting cell proliferation and migration [126,127,128,129,130,131,132,133]. These processes can be modulated by XIST through interaction with miRNAs, as in NSCLC where XIST acts as a sponge for miR-186-5p, and its knockdown suppresses multiplication and invasion, as shown by Hu et al. for cancerous bladder cells [131,134].

The complex roles of XIST in human carcinoma have been recently reviewed by Yang et al. [135].

### 3.2. MALAT1

One of the most studied lncRNAs, MALAT1 is involved in several dysregulations found in cancer and is responsible for coordination of alternative splicing.

This transcript results from a precursor containing a tRNA-like small ncRNA, known as MALAT1-associated small cytoplasmic RNA (mascRNA) after cleavage by RNAse P [136]. It is a highly conserved and extremely abundant lncRNA, therefore it is also known as nuclear-enriched abundant transcript 2 (NEAT2) [20,137]. A distinctive feature of MALAT1 is the presence of a triple helix at the 3’end that stabilizes the structure and replaces the poly(A) tail that is conventionally found in other lncRNAs. This helical element consists of a U-rich internal loop which combines with a downstream A-rich tract, resulting in the protection of this lncRNA molecule end by inhibiting rapid nuclear decay [138].

Little is known so far about this abundant lncRNA’s molecular mechanism of action. MALAT1 has been shown to act either as a sponge or circular endogenous RNA (ceRNA) for miR-195 [39] or as a scaffold for ribonucleoprotein complexes with epigenetic functions heterogeneous nuclear ribonucleoprotein C1/C2 (hnRNP C) [39].

Functionally, MALAT1 is involved in several cellular processes, such as alternative splicing and transcriptional regulation [139]. Being localized in the nuclear speckles—interchromatin granule clusters rich in splicing factors—MALAT1 is a key master regulator of alternative splicing mechanisms [140,141]. In this respect, several studies have confirmed the interaction between MALAT1 and spliceosomal proteins, or proteins rich in serine and arginine (SR proteins) also involved in splicing regulation [139,142].

MALAT1 originates from a gene on chromosome 11 that is transcribed by RNA Pol II. In turn, MALAT1 controls transcription of several genes. By interacting with a member of PRC2 complex- Enhancer of zeste homolog 2 (EZH2), MALAT1 is actively involved in the methylation of histone H3 in lysine 27 (H3K27) and inhibition of tumor suppressor genes, therefore contributing to loss of proliferation control, cell migration, invasiveness, and pro-metastatic programs [139,143,144].

#### 3.2.1. Chemical Modifications in MALAT1

Several chemical modifications are evidenced in MALAT1. An m^6^A modification in position 2515/2577 of the triple helix at the 3’end in MALAT1 is catalyzed by the nuclear METTL16. This interaction with the m^6^A writer has been proven both in vitro and in vivo [36,139], and results in a more flexible and adaptable conformation of the molecule [17,20]. This post transcriptional change of the MALAT1 hairpin consisting in m^6^A methylation is a reversible modification [139] that may alter MALAT1’s interaction with its RNA binding proteins [145]. For instance, m^6^A is able to modulate accessibility and interaction with heterogeneous nuclear ribonucleoprotein G (HPRNPG) [146] or with hnRNP C [39] by enhancing the binding to U5 tract.

Additionally, another methylation occurs in MALAT1 in m^1^A8398, previously described by Gutschner et al. [147]. This chemical modification appears in the T-loop in mascRNA generated after cleavage of RNA precursor [56]. By means of RNA bisulfite conversion and NGS, Squires et al. proposed m^5^C possible methylation sites [73]. In addition, pseudouridine residues were found in positions U5160, U5590, and U3374 [20,95,96]. The roles of these chemical modifications and how they modulate MALAT1 function is currently unknown, and requires further in-depth investigation.

#### 3.2.2. MALAT1 in Cancer

A recent study from Amodio et al. [139] places MALAT1 functions in key spots of the cancer development process, since it regulates transcription of oncogenic targets and it is regulated itself by interaction with transcription factors. This lncRNA can mediate transcription factors binding to target genes promoters, or can act as a sponge to sequester miRNAs, controlling miRNAs suppressor effects on oncogenic targets. On the other hand, epigenetic modifications occurring at histone level—for instance, demethylation of histone H3 in lysine 9 position (H3K9) by a demethylase that binds to the MALAT1 promoter—may result in MALAT1 lncRNA overexpression [139,148].

By regulating gene expression and coordinating splicing, MALAT1 is practically involved in cell cycle and proliferation dysregulation, as well as cell migration and metastasis in several types of cancer [147,149,150,151,152,153,154,155]. For example, MALAT1 function was recently studied in ovarian cancer, where it was shown to promote epithelial to mesenchymal transition (EMT) and a pro-metastatic phenotype, although the mechanism is unknown [17,156]. In brain cancer, renal cell carcinoma, and gastric tumors, MALAT1 acts through a miRNA (miR-155, miR-200, and miR-122, respectively) and represents an overall negative prognostic marker for survival, whereas in pancreatic cancer it targets the Hippo-YAP1 pathway and is informative for an increase in patient survival [139].

Considering its implications in cell proliferation, and generally in cancer progression, MALAT1 is a potential therapeutic target for cancer treatment [146,156,157,158].

### 3.3. HOX Antisense Intergenic RNA

HOX antisense intergenic RNA (HOTAIR) lncRNA is 2.2 kb long and results from the antisense transcription of the HOXC gene cluster [159]. HOTAIR is involved in gene silencing by interaction with two chromatin-modifying complexes [159,160,161]. The 5’ end of HOTAIR interacts with PRC2 and leads to transcriptional silencing across 40 kb around the HOXD locus [160]. The other complex HOTAIR recruits is the histone demethylase complex lysine specific demethylase 1/co-repressor of RE1-silencing transcription factor/RE1 silencing transcription factor (LSD1/CoREST/REST) via the 3’ end region [159,162], to induce methylation of histone H3 at lysine K27 and demethylation of histone H3 at lysine K4 [160]. With a completely different outcome, HOTAIR can interact with a MLL1 methyltransferase and induce histone H3 lysine-4 trimethylation (H3K4me3) that promotes transcription [163,164,165].

#### 3.3.1. Chemical Modifications in HOX Antisense Intergenic RNA

In the vicinity of the LSD1-binding site of HOTAIR, a methylated C was identified at position 1683 in two different cell types in HEK293 and NT2 cell lines. Methylation of C1683 was found to be invariable, regardless of the different levels of HOTAIR expression and the different types of cancer cells, such as Hs578T and BT-20 breast cancer and HOC7 ovarian cancer cell lines. Since methylated C1683 is found in the vicinity of the LSD1-binding site, it has been suggested that it affects the HOTAIR interaction with LSD1 complex [123].

Concerning other types of RNA modifications, in particular m^6^A, the studies conducted so far did not identify an m^6^A site consistent in all cell types investigated. In HOTAIR from HEK293T cells, a single m^6^A peak region near the 5’-end region was identified, but in HepG2 cells and human brain tissue, there was no m^6^A signal detected in HOTAIR [101,102].

#### 3.3.2. HOX Antisense Intergenic RNA in Cancer

HOX antisense intergenic RNA is a lncRNA with numerous roles in cancer development. Altered expression of HOTAIR is found in many types of cancer, and promotes metastasis and tumor invasiveness through epigenetic gene silencing [166,167,168,169,170,171]. Cancer stem cells from breast, oral and colon carcinomas, and gliomas express high levels of HOTAIR associated with increased stemness and metastatic potential [172,173].

High levels of HOTAIR correlated with metastasis and poor prognosis have been found in lung cancer [174], hepatocellular carcinoma [175,176], breast cancer [166], gastric cancer [177,178], colorectal cancer [179], cervical cancer [180], ovarian cancer [181], head and neck carcinoma [182], and esophageal squamous cell carcinoma [183]. Just recently, elevated HOTAIR expression was also identified in adrenocortical carcinoma, and it was demonstrated to be involved in stimulating cell proliferation [184]. In addition, another recent study showed the potential of HOTAIR to promote osteosarcoma development [185].

Evidence supporting HOTAIR’s role in mediating drug resistance has emerged for many types of cancer investigated. Elevated HOTAIR expression was found in samples from drug-resistant patients with NSCLC [186]. Similar results for HOTAIR’s potential to promote resistance to cisplatin or other types of chemotherapy drugs have been obtained for other types of cancer as well, such as hepatocellular carcinoma [187], breast cancer [188], gastric cancer [189], colorectal cancer [173,190], cervical cancer [180], and ovarian cancer [191,192].

### 3.4. Other Long Non-Coding RNAs that Carry Chemical Modifications and May be Involved in Cancer

**Figure 2:**
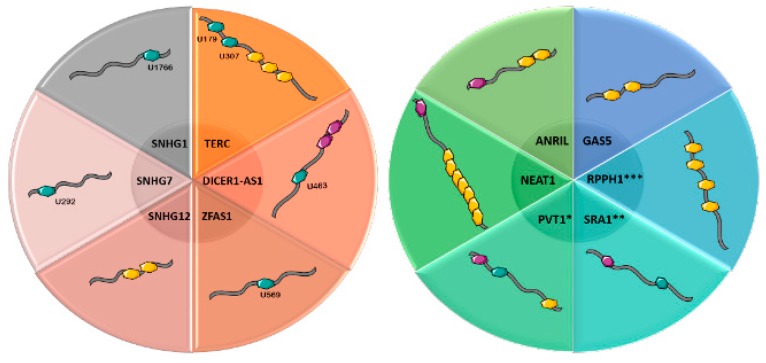
RNA modifications and their positions (where known), occurring in some lncRNAs: TERC, DICER-AS1, ZFAS1, SNHG12, SNHG7, SNHG1, GAS5, RPPH1, SRA1, PVT1, NEAT1, ANRIL. *For these lncRNAs studies have reported different numbers of RNA modification sites (presented in Table 2). Blue hexagon—ψ modification, purple hexagon—m^6^A modification, yellow hexagon—m^5^C modification, pink hexagon—m^1^A modification. There are two separate circles, to present the lncRNAs in a better format.

Apart from the three main lncRNAs discussed in detail so far, other lncRNAs with roles in cancer development are under extended investigation to determine their role and whether or not they also carry RNA modifications (Table 2). A correlation between these lncRNAs and the identified RNA modifications carried is presented in Figure 2.

Some studies have carefully mapped ψ sites in lncRNA (Table 2). These lncRNAs are also involved in malignant processes, with their expression being upregulated or downregulated [154,193,194,195]. Zinc finger antisense 1 (ZFAS1) is one example; this lncRNA has a specific site for uridylation (U569) [117] and is overexpressed in bladder, lung, colon, hepatic, and gastric cancer [172,196] (Table 2). Unfortunately, there is no known link between these two facts. Additional studies are needed in order to ascertain the correlation between ψ modification and cancer involvement.

Small nucleolar RNA host gene (SNHG) 1 and 7 each have one modified uridine residue, SNHG1 in position 1766 and SNHG7 in position 292 [95,96]. While upregulation of SNGH7 inhibits apoptosis and promotes the proliferation of cancerous gastric cells [197], SNHG1’s overexpression is correlated with advanced colorectal cancer stage and tumor recurrence [198]. Tian et al. found that SNHG1 acts as a sponge for miR-145, a well-known tumor suppressor, thus facilitating cancerous cell proliferation [198].

DICER1 Antisense RNA 1 (DICER1-AS1), another lncRNA with a ψ site (U463), is upregulated in osteosarcoma cells, promoting their proliferation, invasion, and autophagy via miR-30b/ATG5 [199].

Telomerase RNA component (TERC) has two modified uridines in its structure as well as three m^5^C [73,117]. Baena-Del Valle et al. recently demonstrated that TERC is overexpressed in all stages of prostatic adenocarcinoma, and that this upregulation is correlated with the expression of proto-oncogene MYC, a known driver of prostate cancer. They found that forced reduction of MYC was associated with low levels of TERC and silencing MYC decreased the activity of TERC promoter, while forced overexpression of MYC resulted in increased levels of TERC. They also discovered MYC in the TERC locus through chromatin immunoprecipitation (ChIP). Knockdown of TERC reduced proliferation of prostate cancerous cells [204].

Squires et al. discovered several m^5^C modifications in antisense non-coding RNA in the INK4 locus (ANRIL), growth arrest-specific transcript 5 (GAS5), nuclear paraspeckle assembly transcript 1 (NEAT1), Pvt1 oncogene non-protein coding (PVT1), ribonuclease P RNA component H1 (RPPH1), SNHG12, and TERC [73]. At the moment, the position of these modified residues has not been carefully mapped. Although each one of these lncRNAs is implicated in various types of human cancer (Table 2), a correlation between their chemical modifications and their involvement in these malignancies is currently unknown. 

ANRIL has two sites for cytosine methylation [73] and is involved in prostate cancer, where it stimulates cell proliferation and migration through the let-7a/TGF-β1/Smad signaling pathway [209]. GAS5 also has two m^5^C positions [73], and it was recently reported by Li et al. that its expression is downregulated in a very aggressive form of breast cancer, specifically triple-negative breast cancer (TNBC). Furthermore, using an ectopic overexpression system, they discovered that upregulation of GAS5 decreased the proliferation rate of TNBC cells and enhanced apoptosis. They concluded that GAS5 competitively binds miR-196a-5p, thus suppressing TNBC progression [211]. Also implicated in breast cancer, RPPH1 inhibits tumorigenesis by downregulating the expression of miR-122 [207].

SNHG12 is involved in both gastric and colorectal cancer, with two currently known m^5^C positions in its structure [73,195,208]. Zhang et al. analyzed the expression of SNHG12 in gastric cells from both tumor samples and adjacent normal tissues, and found that this lncRNA is significantly overexpressed in cancerous cells, promoting cancer progression by acting as a molecular sponge for miR-320. Additional analysis of SGC-7901 and AGS cell lines showed that inhibition of SNHG12 suppressed cell growth, proliferation, colony formation, and invasion [195]. The same tumor promoter role was observed in colorectal cancer cells, where overexpression of SNHG12 facilitates cell growth and inhibits apoptosis [208].

NEAT1 can either be upregulated or downregulated, with aberrant overexpression in pulmonary, oesophageal, colorectal, and hepatocellular carcinoma, and low expression in acute promyelocytic leukaemia (APL), both associated with poor prognosis [205]. Several m^5^C sites have been found for NEAT1 [73]. For PVT1, an important carcinogenic lncRNA, two m^5^C positions have been detected in its structure [73]. High levels of expression positively affect tumor cell proliferation, migration, and invasion, at the same time preventing apoptosis [206].

## 4. Discussion

### Correlation Between Chemical Modifications in lncRNAs and Possible Cancer Implications

Despite the current remarkable evolution of the epitranscriptomics and lncRNA fields of research, little is known about the functional roles of lncRNAs in cancer, or their complete mechanisms of action. In order to advance personalized cancer therapies or pharmacological studies targeting lncRNAs or the signaling pathways they govern, it would be crucial to elucidate the interplay between the dynamics of chemical modifications occurring in lncRNAs, the consequences upon lncRNAs or their targets’ function, and modulation of cancer features in response to these changes. Consequently, a correlation between chemical modifications in lncRNAs and possible cancer implications is further presented.

m^6^A interferes with pluripotency and cellular differentiation, both of which are associated with cancer progression. Modification of m^6^A is described as a potential target for treatment of human cancers, since it has implications in metabolism, stem cell self-renewal, and metastasis. As a result, modulation of metabolism in tumors would be reflected in the regulation of m^6^A. [19].

m^5^C can be found in multiple lncRNAs which also play a role in malignant processes. These lncRNAs can be either upregulated (e.g., ANRIL, SNHG12, NEAT1 in some cancers, PVT1) or downregulated (e.g., GAS5, NEAT1 in APL) in order to promote cell proliferation and migration, and prevent apoptosis of cancerous cells [195,205,206,209]. If their expression is switched and they are inhibited instead of being upregulated (SNHG12), or over-expressed instead of under-expressed (GAS5), they can determine a decrease in cell growth, colony formation, and invasion, and increase apoptosis [195,209].

The connection between ψ and cancer was first reported in studies conducted on urinary metabolites from patients suffering from this disease [212,213]. Being a modified nucleoside, ψ cannot be recycled and is eliminated through urine, with its levels depending on the rate of glomerular filtration and RNA turnover, resulting in higher levels for cancer patients [214]. Because of this, the assessment of urinary ψ levels has been proposed as a potential tumor marker, but it has not been included in routine diagnostics [215].

**Figure 3:**
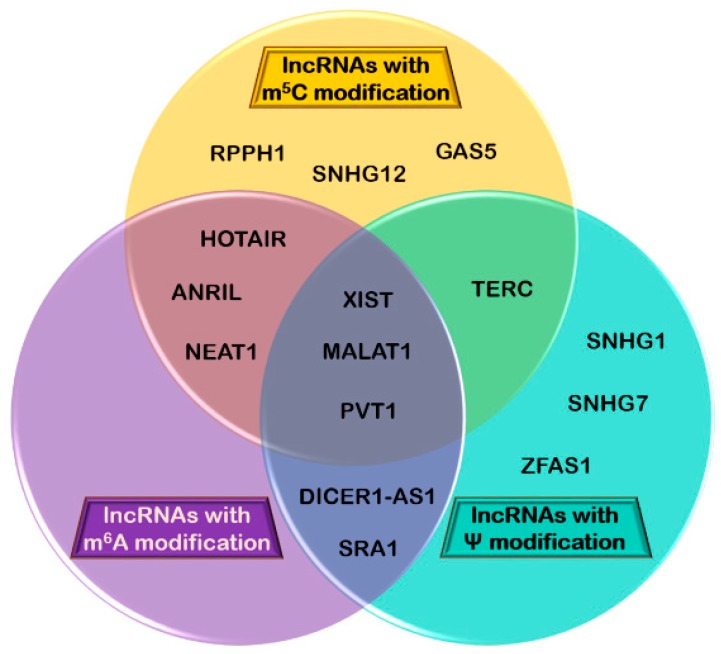
Venn diagram illustrating the overlap between different lncRNAs carrying m^6^A, m^5^C, ψ modifications.

Mutations in PUSs lead to impaired activity of said enzymes, which has been recognized as a potential trigger for cancer in both inherited and sporadic tumors. In breast carcinomas, for example, low dyskerin expression, correlated with reduced rRNA pseudouridylation, promotes neoplastic transformations by suppressing translation of mRNA molecules that code for tumor suppressors. The reason behind this is the localization of the pseudouridylation sites for dyskerin in the ribosome; they are located in specific domains important for tRNA and mRNA binding [216,217]. In contrast to these findings, some have observed that in different types of human cancer (e.g., breast, lung, hepatocellular and prostate carcinomas), dyskerin expression and pseudouridylation levels are frequently higher, and are associated with poor patient prognosis, malignant progression, and lower disease-free survival [218,219,220]. Pseudouridylation has also been found in lncRNAs correlated with cancer, such as XIST and MALAT1, as previously discussed [95,96,117]. Recent studies have shown that lncRNAs can host one or more modifications to modulate their activity. Therefore, we considered it important to illustrate the overlap between the most important cancer-related lncRNAs and major RNA modifications occurring in lncRNAs (Figure 3).

Deregulations have also been found at the level of readers, erasers, and writers of already described chemical modifications, particularly for m^6^A. For instance, in breast cancer some erasers and writers seem to be related to hypoxic condition (ALKBH5), and respectively to a shorter survival of patients as a consequence of a lower expression (METTL14). In hepatocellular carcinoma tissue, a significant low expression of METTL14 has been found and correlated with metastasis inhibition [221].

In glioblastoma tumor, the key m^6^A writers known to have a potential role in tumor course are METTL14 and METTL3, for which the experimental knocking-down expression in cellular models results in tumor cell growth. Moreover, the protein eraser ALKBH5 seems to display a high expression in glioblastoma and sustains tumor cells growth via fork head box M1, thus ALKBH5 deregulation is linked to a poor outcome [222]. It has been shown that silencing either METTL14 or ALKBH5 leads to cancer growth inhibition, a deregulation of the transforming growth factor– β signaling pathway, and epithelial-to-mesenchymal transition. Recently, Panneerdoss et al. have shown that a certain RNA methylation level is required to control the regulation of critical transcripts’ expression; therefore, it is necessary to maintain the thin balance of m^6^A writers–readers–erasers expression in order to prevent tumorigenesis activation and progression [223].

These myriads of ‘switch’ deregulations contribute to setting a therapeutic stage as inhibitors of m^6^A modifications are now deeply explored for altering m^6^A chemical profiles in a drug development context. Meclofenamic acid is such a potential drug example as it is a very selective inhibitor for a certain eraser (FTO), but, although promising, such drugs are not specific for multiple cancers, because of cancer heterogeneity [224,225].

## 5. Conclusions

This review integrates lncRNAs and their function into the cancer development landscape. Once considered ‘junk’ non-coding material, lncRNAs prove to be key regulators of gene expression and molecular events during transcription, and modulators of signaling pathways and post-transcriptional regulation guided by miRNAs. Therefore, lncRNAs have progressed rapidly to a privileged position of control—a second layer of post-transcriptional regulation. Chemical modifications in lncRNAs generally induce a dysregulated state, and convert altered lncRNAs to transcripts potentially critical for cancer progression. In this context, MALAT1, which carries an m^6^A and several pseudouridine modifications, has been reported as a master regulator of metastasis and a potential therapeutic target. MALAT1 has been found to control proliferation, migration, and apoptosis in many different human cancers, and its overexpression has also been correlated with drug resistance. Specific cytosine methylation was found in HOTAIR, which promotes metastasis in several cancer types, but no pseudouridylation was yet reported for this lncRNA. XIST includes all three types of chemical modifications—m^6^A, m^5^C, and ψ— and can act either as an oncogene or as a suppressor in multiple types of cancer. Overexpression of XIST is associated with advanced tumor stage and overall poor prognosis in human cancers. The molecular mechanisms triggered by chemical modifications in lncRNAs that lead to changes in their function are not yet fully understood, and need further molecular characterization to complete another ‘piece of the puzzle’ in the interplay between lncRNAs and cancer.

## Figures and Tables

**Table 1:** The substrates for NOP2/SUN RNA methyltransferase (NSUN) family members.

NSUN1	NSUN2	NSUN3	NSUN4	NSUN5	NSUN6	NSUN7	DNMT2
rRNA	tRNA, mRNA, ncRNA	mt-tRNA	mt-rRNA	rRNA	tRNA	Substrate(s) unknown	tRNA

**Table 2:** Chemical modifications found in lncRNAs and their possible involvement in different types of cancer. *Number of the positions of RNA modifications varies between studies. MALAT1: metastasis associated lung adenocarcinoma transcript 1; HOTAIR: HOX antisense intergenic RNA; XIST: X-inactive specific transcript; ANRIL: antisense non-coding RNA in the INK4 locus; DICER-AS1: DICER1 Antisense RNA 1; NEAT1: nuclear-enriched abundant transcript 2; PVT1: Pvt1 oncogene non-protein coding; SRA1: Steroid Receptor RNA Activator 1; TERC: telomerase RNA component; GAS5: growth arrest-specific transcript 5; RPPH1: ribonuclease P RNA component H1; SNHG: Small nucleolar RNA host gene; ZFAS1: Zinc finger antisense 1.

Chemical Modification	lncRNA	No. of Position(s)	Correlation with Cancer Type	References
m^6^A	MALAT1	2 (A2515, A2577)	Pancreatic, hepatic, and ovarian cancer	[138,154]
	HOTAIR	1 (*)	Gastric, colorectal, pancreatic, hepatic, breast, and skin cancer	[101,166]
	XIST	1–14 (*)	Leukemia, colorectal carcinoma	[101,102,123]
	ANRIL	1 (*)	Prostate cancer	[101]
	DICER1-AS1	2 (*)	Osteosarcoma	[101,199]
	NEAT1	1 (*)	Cervical and non-small cell lung cancer, clear renal cell carcinoma	[102,200,201,202]
	PVT1	1–2 (*)	Gastric, breast, non-small cell lung cancer, hepatocellular carcinoma, glioma	[101,102]
	SRA1	1–4 (*)	Hepatocellular carcinoma	[101,102,203]
m^5^C	MALAT1	7 (*)	Hepatic, pancreatic, ovarian	[73]
	HOTAIR	1 (C1683)	Gastric, colorectal, pancreatic, hepatic, breast and skin cancer	[123,166]
	XIST	5 (C701, C702, C703, C711, C712)	Leukemia, colorectal cancer	[123]
	TERC	3 (*)	Prostate cancer	[73,204]
	GAS5	2 (*)	Breast cancer	[73]
	NEAT1	7 (*)	Lung, oesophageal cancer, colorectal, hepatocellular carcinoma, promyelocytic leukaemia	[73,205]
	PVT1	1–2 (*)	Gastric, breast, non-small cell lung cancer, hepatocellular carcinoma, glioma	[73,109,206]
	RPPH1	1–6 (*)	Breast cancer	[71,73,109,207]
	SNHG12	2 (*)	Colorectal, gastric cancer	[73,195,208]
	ANRIL	2 (*)	Prostate cancer	[73,209]
ψ	MALAT1	3 (U3374, U5160, U5590)	Pancreatic, hepatic and ovarian cancer	[95,96]
	XIST	1 (U11249)	Leukemia, colorectal carcinoma	[96]
	TERC	2 (U179, U307)	Prostate cancer	[117,204]
	SNHG7	1 (U292)	Gastric cancer	[96,197]
	SNHG1	1 (U1766)	Colorectal cancer	[95,198]
	ZFAS1	1 (U569)	Bladder, lung, colon, liver, gastric cancer	[117,196,210]
	DICER1-AS1	1 (U463)	Osteosarcoma	[96,199]
